# Gallbladder volvulus:  a case report

**DOI:** 10.1186/s13256-021-03115-7

**Published:** 2021-10-09

**Authors:** Phillip Croce, Samuel Licata

**Affiliations:** 1 Department of General Surgery, Trinity Health System, Trinity West, Steubenville, OH USA; 2 West Virginia School of Osteopathic Medicine, Lewisburg, WV 24901 USA

**Keywords:** Gallbladder volvulus, Gallbladder torsion, Acute cholecystitis, Gangrenous cholecystitis, Case report

## Abstract

**Background:**

Gallbladder volvulus is a rare pathology first reported by Wendel in 1898. Although the main pathological process associated with gallbladder volvulus is not known, there is clinical evidence suggesting that lack of gallbladder adhesions to the liver leads to an eventual twisting around the cystic bile duct (a process that seems to favor older female populations).

**Case presentation:**

In this report, an 81-year-old Caucasian elderly female presented to the emergency department with acute/severe right upper quadrant pain, which was also accompanied by an elevated leukocyte count. Relevant imaging showed a distended gallbladder with gallbladder wall thickening and a dilated common bile duct. The patient was subsequently admitted to the hospital for acute cholecystitis and scheduled for surgery the next day. Upon laparoscopic surgery, the gallbladder was black and gangrenous with no visible adhesions to the liver. Further inspection demonstrated that the gallbladder had twisted clockwise around the cystic bile duct.

**Conclusions:**

While many previous cases have been reported since Wendel, further case studies are nevertheless important to help guide proper clinic evaluation and pinpoint the potential for a gallbladder volvulus.

## Background

Gallbladder volvulus (or torsion) is a rare pathological process that commonly mimics acute cholecystitis. While the main etiology is undetermined, it has often been reported as originating from a lack of adhesions to the liver (“floating gallbladder”) and a propensity to twist around the cystic bile duct [1, 2]. Furthermore, its prevalence has been primarily noted in elderly patients, with few cases reported in young adults/infants [3]. Diagnosis proves challenging as imaging is consistent with acute cholecystitis (gallbladder wall thickening, gallbladder wall edema, presence of stones/biliary sludge, and so on), with lab studies offering even less of a complete picture (consistent with acute cholecystitis: normal liver enzymes, leukocytosis, and elevated C-reactive peptide) [4, 5]. In such a case, surgical intervention will discover a black gallbladder, with surrounding inflammation to the peritoneum, prompting the need for immediate surgery to untwist the cystic duct and remove the gallbladder altogether. Although an increasing number of cases have been reported since the beginning of the 2000s [3], further information regarding this unique surgical presentation is nonetheless important to guide future diagnoses and surgical procedures.

## Case presentation

**Fig. 1:**
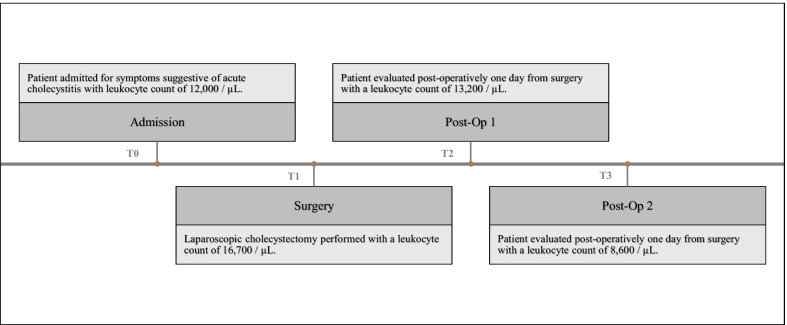
Case timeline. Representation of the case as a timeline, with corresponding leukocyte counts for reference. T0 refers to the day of initial presentation/admission, T1 refers to the day of surgery, T2 refers to the day of postoperative evaluation 1, and T3 refers to the day of postoperative evaluation 2. The timeframe between each interval is approximately 24 hours

An 81-year-old Caucasian female presented to the emergency department with a 1-day history of acute epigastric pain. The pain was described as sharp and severe with radiation to the right upper quadrant, which was associated with nausea but no vomiting, jaundice, fever, diarrhea, constipation, or chills. Although the pain was initially provoked by a fatty meal (“hamburger and soup”), the patient did elaborate that these symptoms had been going on for several years but were unrelated to fatty food consumption. The patient had a past medical history significant for chronic hypertension, arthritis, hypercholesterolemia, hypothyroidism, and depression. Family history was significant for pancreatic cancer in the patient’s mother. Physical examination noted vitals to be within normal limits with pain to palpation in the right upper quadrant, left lower quadrant, and right lower quadrant. A timeline of the events pertaining to the case, as well as the corresponding leukocyte counts for each day, is represented in Fig. 1.

On admission to the hospital, laboratory results were obtained and noted a leukocyte count of 12,000/µL with normal liver enzymes and total bilirubin. Subsequent computed tomography (CT) demonstrated a distended gallbladder with gallbladder wall thickening, and an abdominal ultrasound (US) also showed distention of the gallbladder (as well as a thickened gallbladder) wall with a dilated common bile duct. Based on these findings, the patient was diagnosed with acute cholecystitis and scheduled for a laparoscopic cholecystectomy the following day, while being managed with intravenous saline and Mefoxin. Zofran and hydromorphone were also used as needed for nausea and pain, respectively.

On the day of the surgery, laboratory tests were obtained a second time, which showed an elevated leukocyte count of 16,700/µL and aspartate aminotransferase (AST) of 59 units/L but a normal alanine aminotransferase (ALT) and total bilirubin. During surgery, the patient was placed under general anesthesia in the supine position and prepped for laparoscopic cholecystectomy. A skin incision was made supraumbilically for the insertion of a 12 mm Optiview port. The abdomen was also insufflated with 15 mmHg of carbon dioxide pressure, and a camera was subsequently inserted into the 12 mm port. Intraabdominal visualization showed a necrotic gallbladder with a large amount of bloody ascites and inflammation to the peritoneum. This discovery prompted the conversion to an open cholecystectomy. A skin incision was then made along the right subcostal margin with a 10-blade scalpel. Bovie electrocautery was used to cauterize any bleeding skin edges and assist with dissection. The anterior rectus fascia, the rectus muscle, and posterior fascia were divided using the Bovie as well. An opening was made in the peritoneum so that the surgeon’s hand could be used for blunt dissection/identification of the triangle of Calot. Upon visual inspection and dissection, the gallbladder was noted to be free floating (no adhesions to the liver) and twisted clockwise 360° around the cystic bile duct while being tethered to the cystic bile duct and cystic artery. The gallbladder was consequently untwisted twice and resected. The abdomen was then irrigated with a liter of normal saline solution, and a drain was introduced through a separate stab incision and placed along the liver edge to relieve any additional buildup of bloody fluid within the abdominal cavity.

## Discussion

GV is a rare pathology that has been gaining increasing understanding since it was first reported by Wendel in 1898 [6]. The classification of a GV involves the degree of rotation that occurs. An incomplete torsion is classified as a rotation about the cystic duct of 180°, and a complete torsion is a rotation exceeding 180° [2]. In the current report, the gallbladder was rotated clockwise 360° around the cystic duct, which is consistent with a complete gallbladder torsion. Of the reported cases, the diagnosis of GV predominantly favors elderly patients, with some cases described in young adults and children [1–3]. In adult patients, one study found that the occurrence in female:male ratio was 4:1. This ratio has been reported previously by many other studies as 3:1. This contrasts with the occurrences in children, which was reported as a male:female ratio of 2.5:1 [3].

The exact mechanism of GV is unknown, but several studies have postulated how such a rare pathology could occur. Previously, it has been described that multiple positions for the gallbladder, in relation to the liver, exist; five have been described, and they are as follows: intrahepatic, attached to liver via peritoneum, attached to liver via a complete mesentery, a redundant mesentery that allows the gallbladder to hang, and an incomplete mesentery. Torsions have the highest chance of occurring when the gallbladder is not completely adhered to the liver. In the case of a redundant or incomplete mesentery, there is enough freedom to allow the gallbladder to float in the peritoneum and twist around the cystic duct and cystic artery. Although it is not certain why there is a lack of mesentery surrounding the gallbladder, there seem to be predisposing factors such as age, female sex, and weight loss [3, 7, 8].

Currently, there is no definitive radiological study that can diagnose GV preoperatively. Ultrasound has the propensity to reveal a floating gallbladder and thickening of the gallbladder wall, and CT often only shows enlargement of the gallbladder. In addition to these radiological modalities, there has been some use of magnetic resonance imaging (MRI) and magnetic resonance cholangiopancreatography (MRCP). MRI has been reported to show T1 weight images that can offer indications of necrosis, while MRCP has been used to obtain a noninvasive picture of the cystic duct and its position in relation to other components of the triangle of Calot [4]. The current report demonstrated similar radiological details with a CT that showed a distended gallbladder with wall thickening and US with a dilated common bile duct. Although these studies fall short of offering a conclusive diagnosis for GV, common radiological signs may prove helpful when assessed in conjunction with the classic labs [leukocytosis, elevated C-reactive peptide (CRP), and relatively normal liver contents—normal liver enzymes and total bilirubin] and symptoms of acute cholecystitis [4, 5].

In addition to radiological data, lab studies may also play an important role in diagnosing the evidence of gangrene and eventually the presence of GV. In the present case, laboratory studies taken the day of surgery indicated significant leukocytosis (leukocyte count of 16,700/µL). On the day of surgery, this was postulated to be related to the pathological process of acute cholecystitis with a chance of gangrene. This markedly elevated leukocyte count returned to normal after surgery on postoperative day 2 (Fig. 1). Gangrenous cholecystitis (GC) has been shown to be associated with leukocytosis and elevated levels for CRP. While both indicate the presence of inflammation, CRP has not been reported as a good marker for the severity. On the other hand, elevated white blood cell count (WBC) has been related to the degree of inflammation. Recently, it has been reported that GC tends to have a mean WBC of 13,400/µL [9]. While this may look favorable, studies have found no significant association between leukocytosis and GC [9, 10]. Instead, the correlation between GC and the presence of both leukocytosis and a history of diabetes seems to demonstrate a promising result. Fagan *et al*. reviewed patient data from 1998 to 2001 with the goal of identifying preoperative prognostic factors for the diagnosis of acute cholecystitis. Interestingly, their study showed that patients with a history of diabetes have greater risk for GC when in the presence of a WBC above 15,000/µL [11]. Likewise, another study that used the presence of diabetes, WBC, AST, ALT, and pericholecystic fluid to predict GC. This report showed that these variables have a specificity of 93%, sensitivity of 83%, positive predictive value (PPV) of 71%, and negative predictive value (NPV) of 96% (*P* < 0.001) in detecting GC [12]. Hence, it seems that the aggregation of variables such as leukocytosis, the presence of diabetes, and radiological evidence consistent with acute cholecystitis can offer insight on the prospect of GC and, furthermore, the possibility of GV as a differential.

The need to identify a GV stems from the notion that it is often found intraoperatively with a necrotic or gangrenous presentation [4, 7]. While no current studies have determined which pathology comes first (GV or GC), the need for emergent surgical intervention is unanimous [7]. What seems to be the major source of error is how to diagnose this pathology preoperatively. Above, it was articulated that several variables can be used to identify GC and, hence, allude to GV as a source. Additionally, Kitagawa *et al*. have published potential criteria that could help clinicians make the diagnosis of GV preoperatively. This criterion includes fluid collection demonstrating a floating gallbladder with horizontal positioning, an enhanced cystic bile duct on the right of the gallbladder, and other signs of ischemia/inflammation [13]. Upon diagnosis of a GV, the surgical intervention involves derotation and removal of the gallbladder. While this is often performed laparoscopically, an open removal technique can also be used [7]. Reddy *et al*. have articulated that laparoscopic removal and derotation should be the preferred approach and that this process allows for decompression to prevent bile duct injury [14].

## Conclusions

A volvulus/torsion of the gallbladder is a rare pathology involving an elderly female with weight loss. The presentation mimics acute cholecystitis both on radiological examination and laboratory evaluation. While these modalities are not conclusive by themselves, the summation of their parts allows for the diagnosis of gangrene and possible volvulus of the gallbladder.

## Data Availability

Data sharing is not applicable to this article as no datasets were generated or analyzed during the current case report.

## Abbreviations

GV: Gallbladder volvulus
CT: Computed tomography
US: Abdominal ultrasound
AST: Aspartate aminotransferase
ALT: Alanine aminotransferase
MRI: Magnetic resonance imaging
MRCP: Magnetic resonance cholangiopancreatography
CRP: C-reactive peptide
WBC: White blood cell count
